# Case Report: Two different acromelic dysplasia phenotypes in a Chinese family caused by a missense mutation in *FBN1* and a literature review

**DOI:** 10.3389/fped.2024.1428513

**Published:** 2024-07-15

**Authors:** Fengyan Tian, Xiao Dong, Ruyue Yuan, Xiaohan Hou, Jing Qing, Yani Li

**Affiliations:** Department of Pediatrics, The First Affiliated Hospital of Zhengzhou University, Zhengzhou, China

**Keywords:** acromelic dysplasia, acromicric dysplasia, geleophysic dysplasia, *FBN1*, recombinant human growth hormone

## Abstract

**Background:**

Acromelic dysplasia caused by *FBN1* mutation includes acromicric dysplasia (AD), geleophysic dysplasia 2 (GD2), and Weill-Marchesani syndrome 2 (WMS2). All three diseases share severe short stature and brachydactyly. Besides phenotypic similarity, there is a molecular genetic overlap among them, as identical *FBN1* gene mutations have been identified in patients with AD, GD2, and WMS2. However, no family with different acromelic dysplasia phenotypes due to the same variant has been described in English reports.

**Case report:**

The proband presented with typical facial features, severe short stature, short limbs, stubby hands and feet and radiological abnormalities. Her elder sister and mother had similar physical features. In addition, her elder sister was found to have aortic valve stenosis by echocardiography. Mutation analysis demonstrated a heterozygous missense mutation, c.5179C>T (p.Arg1727Trp) in exon 42 of the *FBN1*. The proband and her mother were diagnosed with AD, and her elder sister with GD2. The proband was treated with recombinant human growth hormone (rhGH) and had a body length gain of 0.72 SDS in half a year.

**Conclusion:**

These findings expand the phenotypic spectrum of *FBN1* gene mutations and highlight that identical *FBN1* genotypes can result in different phenotypes of acromelic dysplasia in a family. The efficacy of rhGH therapy in patients with acromelic dysplasia is controversial. More follow-up is needed on the long-term efficacy of rhGH therapy.

## 1 Introduction

Acromelic dysplasia caused by fibrillin 1 (*FBN1*) gene mutation is an autosomal dominant disorder, consisting of acromicric dysplasia (AD, OMIM #102370), geleophysic dysplasia 2 (GD2, OMIM #614185), and Weill-Marchesani syndrome 2 (WMS2, OMIM #608328) (1). AD, with an approximate prevalence of <1 in 1,000,000, is a rare skeletal dysplasia characterized by severe short stature, small hands and feet, stiff joints, thickened skin, and progressive dwarfism with normal birth length and intelligence (2). Distinct facial features include a round face, long eyelashes, a bulbous nose with anteverted nostrils, a long and prominent philtrum, and thick lips with a small mouth (3). Radiological characteristics include delayed bone age, cone-shaped epiphyses, short metacarpals with internal notch of the second metacarpal and external notch of the fifth metacarpal, internal notch of the proximal femoral epiphyses, and ovoid vertebral bodies (4). GD2 is distinct from AD by characteristic happy facial features, progressive cardiac valvular disease, tracheal stenosis, severe and recurrent respiratory problems, hepatomegaly, and tiptoe gait, among which the cardiorespiratory issues are usually responsible for poor prognosis (5). WMS2 is distinguished from AD and GD2 by ocular involvement, such as spherophakia, ectopia lentis, myopia, glaucoma, and cataract (6). Heterozygous mutations located in exons 41 and 42 encoding the transforming growth factor (TGF)-β-binding protein-like domain 5 (TB5) of *FBN1* and disturbance of the TGF-β signaling pathway have been suggested as the potential mechanisms of AD and GD2 (4). Identical mutations of the *FBN1* gene have been identified in AD, GD2, and WMS2 from different countries, indicating a molecular genetic overlap among them (7–9). In Chinese literature, seven members of a three-generation Chinese family were reported to have both mild GD2 and AD phenotypes caused by *FBN1* gene mutation c.5099A>G (p.Tyr1700Cys) (10). However, no similar phenomenon has been described in English reports to date. Here, we report two different acromelic dysplasia phenotypes (AD and GD2) in three members from a Chinese family associated with a missense mutation in exon 42 of the *FBN1* gene c.5179C>T (p.Arg1727Trp). In addition, the clinical and genetic characteristics of different phenotypes of acromelic dysplasia due to identical *FBN1* gene mutations and the efficacy of recombinant human growth hormone (rhGH) therapy in acromelic dysplasia are summarized.

## 2 Case report

### 2.1 Patient 1

The proband was a 2-year-old Chinese girl. She was admitted for short stature and slow postnatal growth. The patient was the third birth of three, born by full-term cesarean section following an uneventful pregnancy of non-consanguineous, with a birth weight of 3,250 g and body length of 50 cm. Progressive growth retardation was noted since she was born, while the mental and motor development was unremarkable. Except for recurrent respiratory tract infections, she had no history of cardiac, hepatic, optic, or nervous system diseases. Family history reported short statures of her father (160.0 cm, −2.08 SDS), mother (141.0 cm, −3.63 SDS), and 15-year-old sister (143.5 cm, −2.96 SDS), while her 11-year-old brother was of normal height (145.0 cm, −0.57 SDS). Furthermore, her paternal grandfather (170.0 cm, −0.44 SDS) and grandmother (155.0 cm, −1.04 SDS) and maternal grandfather (171.0 cm, −0.28 SDS) and grandmother (162.0 cm, 0.26 SDS) were healthy and of normal height.

Physical examination found her body length was 78.5 cm (−3.11 SDS) and body weight was 9.0 kg (−3.09 SDS). She had an upper segment-to-lower segment ratio of 1.62 (+0.90 SDS). Her arm span was 71.0 cm. She presented with a round face, long eyelashes, a bulbous nose with anteverted nostrils, prominent philtrum, thick and small lips, and stubby hands and feet, without thickened skin or joint stiffness. No hoarseness or cardiac murmur was noted. The liver and spleen were impalpable.

**Figure 1:**
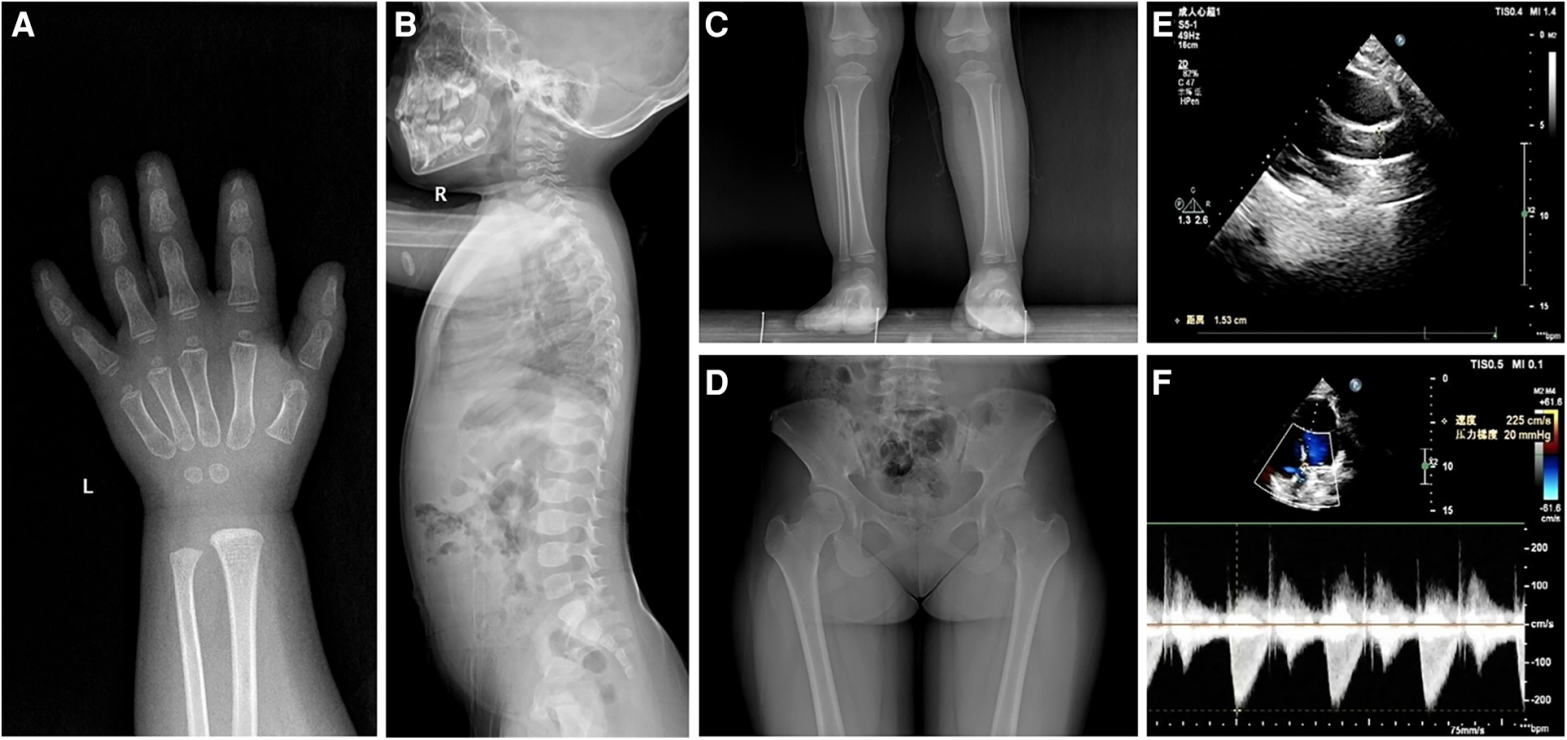
Radiological images showing (**A**) the internal notch in the second metacarpal and the external notch in the fifth metacarpal, (**B**) ovoid vertebral bodies, (**C**) cone-shaped epiphysis of the left leg in patient 1, and (**D**) hip dysplasia and the internal notch of the femoral heads in patient 2. Ultrasound images showing (**E**) aortic valve stenosis with an inner diameter of 15 mm and (**F**) a mild increase in aortic outflow of 2.2 ms^−1^ in patient 2.

Except for insulin-like growth factor (IGF)-1 at a normal low value of 49.8 ng/ml (normal 49.0–289.0 ng/ml), baseline investigations showed no obvious abnormalities in hematology, biochemistry, thyroid function, IGF binding protein-3, and 25-hydroxyvitamin D3. Karyotype analysis showed 46, XX, SRY-negative. Imaging examinations indicated 2-year-old bone age, brachydactyly, the internal notch on the proximal end in the second metacarpal and the external notch on the proximal end in the fifth metacarpal in left-hand radiography, ovoid vertebral bodies in spinal column lateral radiography, and cone-shaped epiphysis in lower extremities radiography (Figures 1A–C). In addition, plain pelvic radiography, echocardiography, abdominal ultrasound, pituitary magnetic resonance imaging, and ophthalmic examination appeared to be normal.

### 2.2 Patient 2

She was a 15-year-old girl and was the elder sister of the proband. She was born by full-term cesarean section with a birth weight of 3,050 g and body length of 50 cm. She suffered from elbow dislocation twice before 3 years old. Her height was 143.5 cm (−2.96 SDS) and weight was 52.0 kg (+0.31 SDS). She had an upper segment-to-lower segment ratio of 1.31 (+2.50 SDS). Her arm span was 132.5 cm. She presented with full cheeks, long eyelashes, a shortened and bulbous nose, a long flat philtrum, thick lips, and stubby hands and feet. Plain pelvic radiography showed hip dysplasia and the internal notch of the femoral heads (Figure 1D). The echocardiography displayed aortic valve stenosis with an inner diameter of 15 mm and a mild increase in aortic outflow of 2.2 ms^−1^ (Figures 1E,F). The abdominal ultrasound revealed no significant findings. No ophthalmic abnormality was observed.

### 2.3 Patient 3

She was a 33-year-old woman and was the mother of the proband. Her height was 141.0 cm (−3.63 SDS). She had an upper segment-to-lower segment ratio of 1.40 (+3.67 SDS). Her arm span was 133.3 cm. She presented with similar unique facial features to the proband, such as a round face, a bulbous nose, prominent philtrum, and thick lips. She also had small hands and feet. She had no joint stiffness, organomegaly, or eye problems. Cardiac and abdominal ultrasound were unremarkable.

### 2.4 Genetic analyses

**Figure 2:**
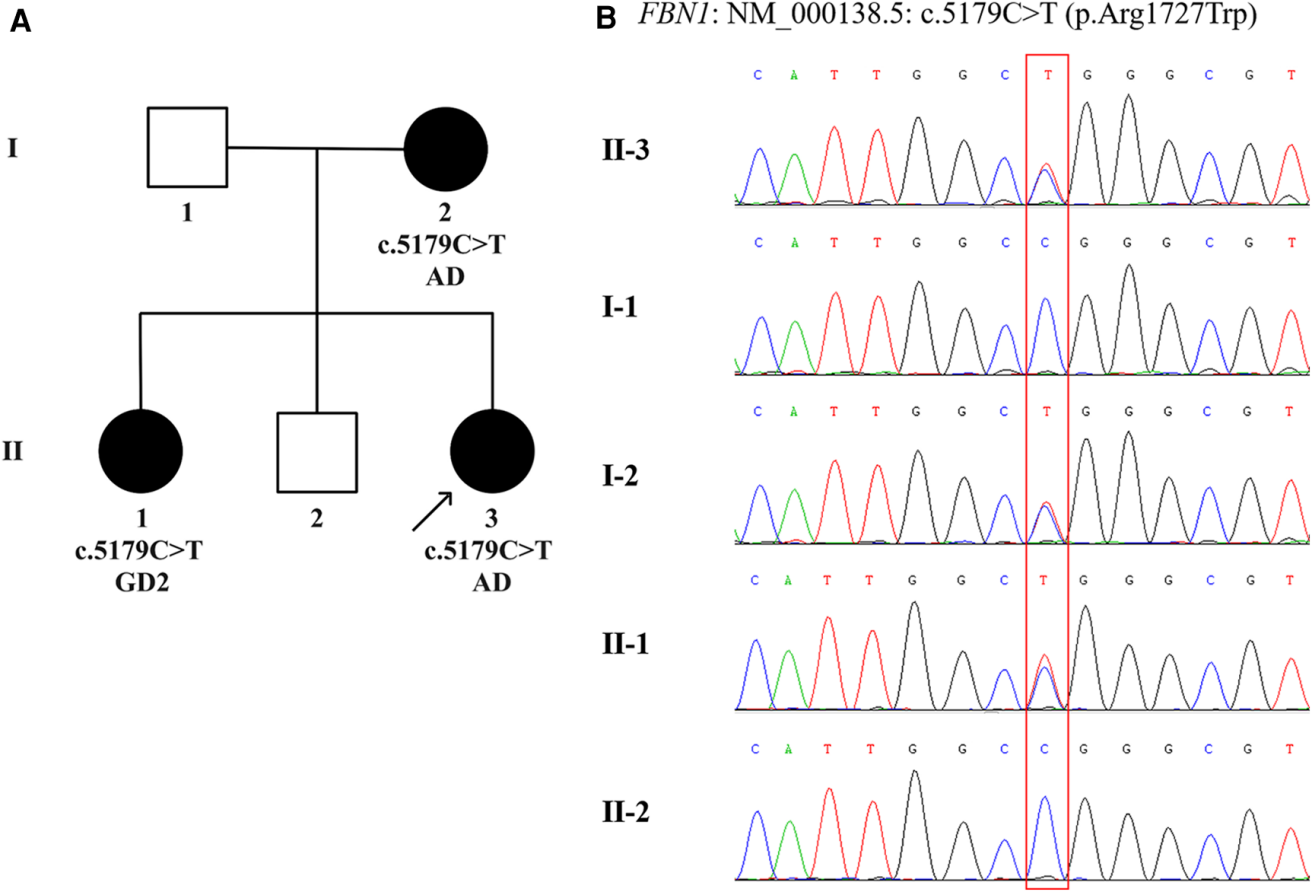
The family pedigree and sanger sequencing chromatograms. (**A**) Pedigree chart of the family with acromelic dysplasia. The dark circles (female) indicate the individuals with acromelic dysplasia carrying a heterozygous *FBN1* mutation. The white squares (male) indicate the individuals who do not carry a heterozygous *FBN1* mutation. The dark arrow below the patient denotes the proband. (**B**) Sanger sequencing confirms a heterozygous missense mutation, c.5179C>T (p.Arg1727Trp) in exon 42 of the *FBN1* (NM_000138.5), in Ⅰ.2, Ⅱ.1, and Ⅱ.3, indicated by the red square. AD, acromicric dysplasia; GD2, geleophysic dysplasia 2.

Given that the proband, her elder sister and mother were all present with typical facial features, significant short stature and skeletal abnormalities (Supplementary Figure S1 and Table S1), DNA extraction and variant analysis were performed. Peripheral blood samples were collected from the proband and her family members in our hospital. Genomic DNA was isolated from the peripheral blood of the proband using a DNA extraction kit (Omega, CA, United States). Whole exome sequencing was performed and enriched for exonic sequences using an Agilent SureSelect XT Human All Exon 50 Mb kit (Santa Clara, CA, United States). Library quality was assessed using Qubit 4.0 (Thermo FisherScientific Inc., USA). The variant was verified by Sanger sequencing combined with polymerase chain reaction in the proband's parents and siblings. Paired-end sequencing was performed using an Illumina sequencing platform (Illumina, San Diego, CA, United States). Following sequencing, data processing and variant annotation were performed using standard analyses. High-quality reads were mapped to the human reference genome GRCh37/hg19. The pathogenicity of the gene variation was determined according to American College of Medical Genetics and Genomics guidelines. The results identified a heterozygous missense mutation in exon 42 of the *FBN1* gene (NM_000138.5): c.5179C>T (p.Arg1727Trp) characterized by autosomal dominant inheritance pattern in the proband, her mother, and elder sister, while neither her father nor elder brother was affected (Figure 2). The genotype, predicted to be of uncertain significance according to the American College of Medical Genetics and Genomics guidelines (PM1 + PM2_S + PP2 + PP3), was co-segregated with the phenotypes among the proband and family members. According to this specific mutation locus, the proband and her mother were finally diagnosed with AD, while her sister was diagnosed with GD2 due to cardiac valvular abnormalities.

### 2.5 Treatment and follow-up

The proband was prescribed long-acting rhGH treatment (0.2 mg/kg/week) at the age of 2 years and 4 months. The body length at the beginning of therapy was 78.5 cm (−3.33 SDS). At a follow-up of 6 months, she had a roughly good response and the body length was 84.5 cm (−2.61 SDS). The rhGH therapy improved growth velocity to about 6.0 cm in half a year, with a body length gain of 0.72 SDS, and IGF-1 was identified to increase to 135.0 ng/ml.

### 2.6 Literature review

Taking “*FBN1*” AND “acromelic dysplasia” in English or Chinese as the keywords, literature was searched at PubMed, CNKI, and Wanfang up to April 2024. The combination of the cases we identified and previous case reports corresponded to 7 *FBN1* variants that contributed to different phenotypes of acromelic dysplasia (1, 2, 4, 5, 7–18). All genetic and phenotypic data are summarized in Table 1. To our knowledge, cases of different acromelic dysplasia phenotypes in a single family due to the same *FBN1* variant have been reported in Chinese literature previously (10), but not yet in English literature.

**Table 1:** Summary of identical mutations in *FBN1* related to different phenotypes of acromelic dysplasia.

cDNA change	Amino acid change	Exon	Protein domain	ACMG classification	Phenotype	References
c.5096A>G	p.Tyr1699Cys	42	TB5	Likely pathogenic	AD GD2	(4) (4, 7, 8)
c.5099A>G	p.Tyr1700Cys	42	TB5	Likely pathogenic	AD GD2	(4, 7, 10, 14) (10)
c.5179C>T	p.Arg1727Trp	42	TB5	Uncertain significance	AD GD2	(13); Present study Present study
c.5182G>A	p.Ala1728Thr	42	TB5	Likely pathogenic	AD GD2	(4, 7, 14) (4)
c.5183C>T	p.Ala1728Val	42	TB5	Likely pathogenic	AD GD2	(1, 2, 7, 15, 18) (4)
c.5243G>T	p.Cys1748Phe	43	TB5	Likely pathogenic	AD GD2 WMS2	(7) (16) (9, 17)
c.5284G>A	p.Gly1762Ser	43	TB5	Likely pathogenic	AD GD2	(5, 7, 8, 14, 18) (4, 5, 8, 11, 12)

*FBN1*: NM_000138.5.

ACMG, American College of Medical Genetics and Genomics; AD, acromicric dysplasia; GD2, geleophysic dysplasia 2; TB5, transforming growth factor-β-binding protein-like domain 5; WMS2, Weill-Marchesani syndrome 2.

In addition, a total of 8 reports about 15 individuals with acromelic dysplasia caused by *FBN1* gene mutation treated with rhGH were retrieved (2, 8, 10, 13, 15, 19–21). The effects of rhGH therapy in *FBN1*-related acromelic dysplasia are summarized in Table 2.

**Table 2:** Summary of rhGH therapy efficacy in *FBN1*-related acromelic dysplasia: present study and other reported cases.

Cases	Gender	Diagnosis	*FBN1* genotype	rhGH therapy			References
				Onset age	Duration (dose)	Efficacy	
1	Male	AD	c.5183C>T	4 years	5 years (0.12–0.15 IU/kg/day)	1st year: 90.5 cm (−3.64 SDS) → 99.5 cm (−2.88 SDS) 2nd–5th year: 99.5 cm (−2.88 SDS) → 117.1 cm (−3.09 SDS)	(2)
2	Male	AD	c.5179C>T	5 years	7 years (0.035 mg/kg/day)	Improved growth velocity to ∼7.0 cm/year A height gain of 1.6 SDS (−3.3 SDS → −1.7 SDS)	(13)
3	Male	AD	c.5243G>A	8 years	Until reporting (NA)	No increase in bone growth	(19)
4	Male	AD	c.5282C>T	10 years	3.5 years (0.1 IU/kg/day)	Improved growth velocity to ∼5.4 cm/year A height gain of 0.98 SDS (−3.21 SDS → −2.23 SDS)	(20)
5	Male	AD	c.5183C>T	10 years and 8 months	5 years and 4 months (0.05–0.066 mg/kg/day)	Initial height 115.0 cm (−3.9 SDS) → adult height 144.5 cm (−4.5 SDS)	(15)
6	Female	AD	c.5183C>T	8 years and 9 months	Ⅰ. IGF-1:3 month Ⅱ. IGF-1 + Leuprolide: 4 months (IGF-1 ≤ 0.09 mg/kg/day)	Ⅰ. Apparent growth velocity increase Ⅱ. Growth velocity decreased to 4–5 cm/year	(15)
7	Female	GD2	c.5086T>G	Ⅰ. 6 years Ⅱ. 8 years	Ⅰ. 4 months (0.03 mg/kg/day) Ⅱ. 6 months (0.06 mg/kg/day)	Ⅰ. A height gain of 0.8 cm Ⅱ. A height gain of 1.5 cm	(21)
8	Male	GD2	c.5243G>C	Ⅰ. 4 years Ⅱ. 6 years	Ⅰ. 5 months (0.03 mg/kg/day) Ⅱ. 3 months (0.06 mg/kg/day)	Ⅰ. No catch-up growth (0.5 cm) Ⅱ. No catch-up growth (0 cm)	(21)
9	Male	GD2	c.5284G>A	6.1 years	3 years (0.3 mg/kg/w)	1st year: a height gain of 0.2 SDS 2nd year: a height gain of 0.2 SDS 3rd year: a height gain of 0.0 SDS	(8)
10	Male	AD	c.5284G>A	4.5 years	1.5 years (0.3 mg/kg/w)	Poor response A height gain of 7.0 cm	(8)
11	Female	AD	c.5099A>G	14 years and 6 months	16 months (1.1–1.4 IU/kg/w) (+15 months Triptorelin +9 months Stanozolol)	Adult height 126.0 cm A height gain of 6.1 cm	(10)
12	Female	AD	c.5099A>G	12 years and 7 months	32 months (1.1–1.4 IU/kg/w) (+53 months Triptorelin +18 months Stanozolol)	Improved growth velocity to ∼3.8 cm/year A height gain of 14.7 cm (0.95 SDS)	(10)
13	Male	GD2	c.5099A>G	8 years and 4 months	41 months (1.1–1.4 IU/kg/w)	Improved growth velocity to ∼4.7 cm/year A height gain of 13.4 cm (0.57 SDS)	(10)
14	Female	GD2	c.5099A>G	5 years and 3 months	41 months (1.1–1.4 IU/kg/w)	Improved growth velocity to ∼5.8 cm/year A height gain of 16.5 cm (0.65 SDS)	(10)
15	Male	AD	c.5099A>G	4 years and 6 months	23 months (1.1–1.4 IU/kg/w)	Improved growth velocity to ∼6.7 cm/year A height gain of 12.6 cm (0.41 SDS)	(10)
16	Female	AD	c.5179C>T	2 years and 4 months	6 months (0.2 mg/kg/w)	Half year: 78.5 cm (−3.32 SDS) → 84.5 cm (−2.61 SDS)	Present study

AD, acromicric dysplasia; GD2, geleophysic dysplasia 2; IGF-1, insulin-like growth factor-1; NA, not available; rhGH, recombinant human growth hormone; SDS, standard deviation score.

## 3 Discussion

The *FBN1* gene, located on the long arm of chromosome 15 (15q15-q21.1), comprises 66 exons and encodes a 2,871-aa (350 kDa) structural protein called fibrillin-1 (22). Fibrillin-1, a large cysteine-rich glycoprotein, consists of 47 epidermal growth factor-like domains, 2 hybrid domains as well as 7 TGF-β-binding protein-like domains, which contain 8 cysteine residues linked by 4 disulfide bonds, essential for domain structure (23). Fibrillin-1 is the primary structural component of extracellular matrix microfibrils present in elastic and non-elastic connective tissues (9). In the extracellular matrix of the growth plate, the microfibrillar network regulates the bioavailability and activity of the TGF-β, a key signaling pathway that modulates linear growth in chondrogenesis and osteogenesis (24).

Mutations in the *FBN1* gene can result in two opposite skeletal features: Marfan syndrome (MFS, OMIM #154700), characterized by tall stature and arachnodactyly, and acromelic dysplasia, characterized by short stature and brachydactyly (8). Acromelic dysplasia caused by *FBN1* mutations is a heterogeneous group divided into AD, GD2, and WMS2. These three disorders share common clinical features, such as short stature, short extremities, short hands and feet, stiff joints, thickened skin, mild facial anomalies, and normal intelligence, as well as some radiological manifestations, including delayed bone age, shortened long tubular bones, cone-shaped epiphyses, and ovoid vertebral bodies (7, 9). The parameters at birth are always normal and the growth retardation is postnatal and progressive. The severity of the short stature is always less than −3 SDS (14). Despite overlapping characteristics, the three distinct entities are distinguished by their unique features. GD2 presents with distinct facial features defined by a happy face with full cheeks, a shortened nose, hypertelorism, a long flat philtrum, and a thin upper lip, and is marked by abnormalities of the cardiorespiratory system, including progressive cardiac valvular thickening, tracheal stenosis, respiratory insufficiency, and hepatomegaly, whereas individuals resembling GD2 but without cardiac valvular or hepatic abnormalities are diagnosed with AD (21). WMS2 can be differentiated by the presence of ocular abnormalities including ectopia lentis, microspherophakia, severe myopia, cataracts, and glaucoma (6). What's more, the identified genotype of AD, GD2, or WMS2 may be associated with diverse phenotypes or with mixed phenotypes (8). Therefore, the clinical presentation of *FBN1*-related acromelic dysplasia is heterogeneous and variable, posing challenges for accurate diagnosis and clinical management. Targeted next-generation sequencing will make it possible to study the spectrum of *FBN1* gene mutations efficiently and comprehensively, providing reference points for early screening and genetic counseling of acromelic dysplasia (17). In addition, comprehensive cardiovascular and ophthalmological assessments are essential in the diagnosis of patients with *FBN1* variants.

In our study, a heterozygous missense mutation c.5179C>T (p.Arg1727Trp) in exon 42 of the *FBN1* gene was detected in the proband, her elder sister and mother. This variant has previously been identified in six members of a three-generation family associated with AD (13). However, it has not yet been identified in GD2 patients. In the present case, not only the AD phenotype but also a novel GD2 phenotype was found in a family carrying the same *FBN1* variant. The proband exhibited severe short stature and facial features, as did her mother and elder sister. Her elder sister also had aortic valve stenosis on echocardiography with a stable heart condition. Complete segregation of the genetic variant was present among the proband and her family members. The clinical findings supported the diagnosis of AD for the proband and her mother, while GD2 for her elder sister. Although both the proband and her 33-year-old mother were diagnosed with AD, her mother didn't even have any significant abnormalities on ultrasound or X-ray. These findings suggest that there is a molecular genetic overlap in acromelic dysplasia groups, as identical *FBN1* genotypes can lead to different acromelic dysplasia phenotypes not only in different countries but also within a family (Table 1). Furthermore, the symptoms of a phenotype induced by the same *FBN1* variant can vary greatly between individuals, even between family members.

Due to the heterogeneity of *FBN1* variation, the mechanism of genotype-phenotype association remains unclear. Disorganization of the microfibrillar network and enhanced TGF-β signaling caused by *FBN1* mutations can trigger either MFS or acromelic dysplasia (25). Mutations in *FBN1* leading to MFS exist along the entire length of the gene (23), while missense mutations clustered in hotspot regions of exons 41 and 42 encoding the TB5 domain of *FBN1* have been identified as causative mutations for short stature (transcript NM_000138.4) (4). Interestingly, mutations in the *FBN1*-TB5 domain can also result in MFS. A systematic review of patients with *FBN1*-TB5 mutations revealed that acromelic dysplasia was caused exclusively by in-frame amino acid substitutions, instead, truncating mutations in the *FBN1*-TB5 have only been reported in MFS (8). Consistent with previous findings, all *FBN1* variants listed in Table 1 were located in the TB5 domain of exons 42 or 43 (unified transcript NM_000138.5 for all data). The exon difference between the data of Table 1 and previous studies lies in the transcripts used. These results illustrated the complexity of the TGF-β signaling pathway with various levels of regulation and suggested that the positions and types of variation might contribute to the clinical phenotypes (4, 8). Further confounding genotype-phenotype correlations, different or even identical *FBN1* mutations are associated with distinct phenotypes classified as AD, GD2, or WMS2. A study found that *FBN1* missense variants involving a cysteine were significantly more likely to cause heart valve disease, and variants that removed a cysteine were associated with a severe phenotype with life-threatening complications (7). Probably unknown disease-modifying factors caused the phenotypic divergence (5).

There is currently no consensus on the surveillance or management of patients with acromelic dysplasia. Symptomatic therapy requires a coordinated multidisciplinary approach involving genetic, pediatric, cardiac, pulmonary, orthopedic, and ophthalmic specialists, with organized follow-up throughout the whole life (7). As for short stature, no large cohort studies have been conducted to evaluate the therapeutic response to rhGH in patients with acromelic dysplasia. Only a few cases with conflicting outcomes have been reported (21). We compared the effects of rhGH therapy in patients diagnosed with *FBN1*-related acromelic dysplasia in Table 2. Some patients showed a good response to rhGH therapy, reporting improved growth rate and height (2, 10, 13, 20). Other patients showed limited benefit from such rhGH-boosting treatment and no significant degree of catch-up growth (8, 19, 21). In our case, the proband's body length increased by 0.72 SDS in 6 months, indicating that rhGH treatment was effective in the short term. To some extent, the curative effect may be influenced by the age of initiation, dosage, duration, and compliance with rhGH. Since the response data of rhGH therapy are insufficient and contradictory, the clinical effect of rhGH therapy remains uncertain. Therefore, long-term follow-up is necessary to verify its efficacy (2). Surgical valve replacement should be considered in GD2 patients who have indications. Note that GD2 patients with cardiac involvement may not develop cardiac symptoms at least until their second decade when the cardiac valvulopathy has reached an advanced stage, indicating that cardiac valvular disease in seemingly stable forms of GD2 tends to be progressive (26). Non-progressive valve defects were detected at an average age of 2.8 years (from 1 to 10 years) in a clinical study (7). It is unknown whether the proband will suffer from heart problems in the future since she was only 2 years old to date, highlighting the need for regular cardiac follow-up for both the proband and her elder sister. Orthopedic complications with progressive joint limitation may develop in patients with AD and GD2. It is recommended that orthopedic examinations be carried out every two to three years in adulthood to provide joint-preserving treatment, particularly for carpal tunnel syndrome and hip dysplasia (14). Losartan has the potential to treat the extracellular matrix defect in GD2 caused by *FBN1* mutations, however, further preclinical animal studies and clinical trials are needed to determine which disease manifestations are likely to be improved by using losartan (12).

In the present study, both AD and novel GD2 phenotypes were found in a Chinese family carrying *FBN1* variant c.5179C>T (p.Arg1727Trp), which had not been identified in GD2 patients in previous reports. The proband was prescribed long-acting rhGH treatment and had a good response in 6 months. Limitations included the inaccessibility of the clinical characteristics of the maternal grandparents, and the short follow-up period of the proband and her elder sister. Long-term efficacy of rhGH in the proband and long-term cardiac function in the proband and her elder sister require long-term follow-up.

## 4 Conclusion

In summary, this article describes the clinical and genetic features of a Chinese family with both AD and GD2 phenotypes induced by the *FBN1* variant c.5179C>T (p.Arg1727Trp), expanding the phenotypic spectrum of the *FBN1* gene mutation. Identical *FBN1* genotypes can result in different phenotypes of acromelic dysplasia not only in different countries, but also within a family (such as AD and GD2). Unknown disease-modifying factors may be responsible for this phenotypic divergence. The efficacy of rhGH therapy in patients with acromelic dysplasia is controversial. Large cohort studies and long-term follow-ups of acromelic dysplasia patients are needed to clarify the curative effect of rhGH treatment.

## Data Availability

The datasets presented in this study can be found in online repositories. The names of the repository/repositories and accession number(s) can be found in the article/**Supplementary Material**.
