# Supplementary material for: Case Report: Two different acromelic dysplasia phenotypes in a Chinese family caused by a missense mutation in FBN1 and a literature review
Source: Front Pediatr. 2024 Jul 15;12:1428513. doi: 10.3389/fped.2024.1428513 (PMC11284092; doi:10.3389/fped.2024.1428513)
Supplement: Supplementary file 1 [file Datasheet1.docx]

Supplementary Material

# Supplementary Figure and Table

## Supplementary Figure

**
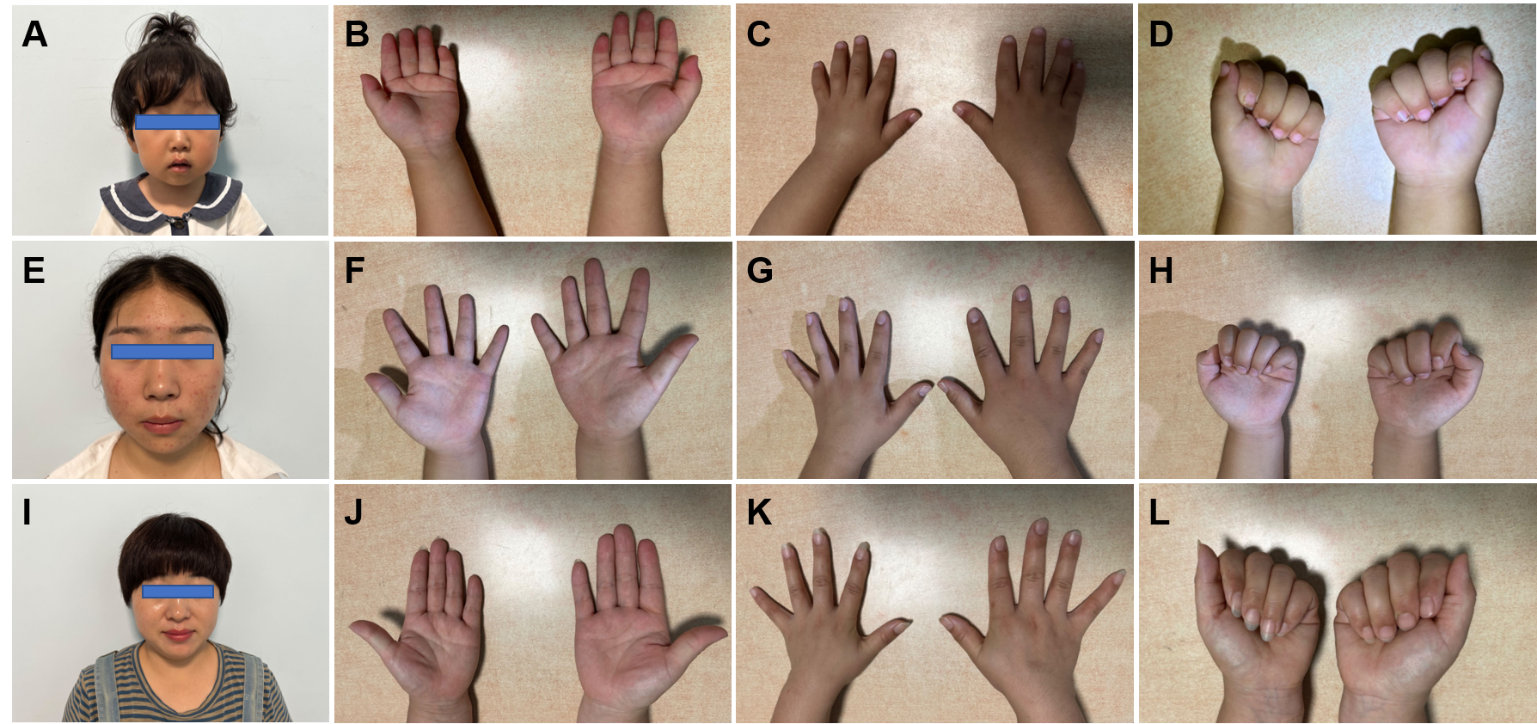
**

**Supplementary Figure 1.** Clinical photos of (A-D) the proband, (E-H) her elder sister and (I-L) mother showing distinct facial features, stubby hands, brachydactyly, without thickened skin or wrist stiffness.

## Supplementary Table

**Supplementary Table 1.** Summary of the clinical phenotypes of the proband, her elder sister and her mother.

| Clinical phenotypes | Proband | Elder sister | Mother |
| --- | --- | --- | --- |
| Diagnosis | AD | GD2 | AD |
| Short stature | + | + | + |
| Short extremities | + | + | + |
| Short hands and feet | + | + | + |
| Brachydactyly | + | + | + |
| Stiff joints | - | - | - |
| Thickened skin | - | - | - |
| Round face | + | - | + |
| Happy face with full cheeks | - | + | - |
| Long eyelashes | + | + | - |
| Bulbous nose | + | + | + |
| Anteverted nostrils | + | - | - |
| Long and prominent philtrum | + | - | + |
| Long and flat philtrum | - | + | - |
| Thick lips | + | + | + |
| Small mouth | + | - | - |
| Radiological features | Internal notch in the 2^nd^ metacarpal and external notch in the 5^th^ metacarpal  Ovoid vertebral bodies  Cone-shaped epiphysis | Hip dysplasia  Internal notch of the femoral heads | - |
| Cardiac valvular abnormalities | - | Aortic valve stenosis | - |
| Tracheal stenosis | - | - | - |
| Respiratory insufficiency | - | - | - |
| Hepatomegaly | - | - | - |
| Tiptoe gait | - | - | - |
| Ocular abnormalities | - | - | - |

Abbreviations: AD, acromicric dysplasia; GD2, geleophysic dysplasia 2.
